# Seasonality at the equator: isotope signatures and hormonal correlates of molt phenology in a non-migratory Amazonian songbird

**DOI:** 10.1186/s12983-018-0284-7

**Published:** 2018-10-29

**Authors:** Rene Quispe, Elizabeth Yohannes, Manfred Gahr

**Affiliations:** 1 Department of Behavioural Neurobiology, Max Planck Institute for Ornithology, Eberhard-Gwinner-Strasse, Seewiesen, 82319 Germany; 2 Stable Isotope Lab, Limnological Institute, University of Constance, Konstanz, Germany; 3 Present address: Departamento Biología Marina, Facultad Ciencias del Mar, Universidad Católica del Norte, Coquimbo, Chile

**Keywords:** Annual timing, Corticosterone, Foraging, Life history stages, Reproduction, Neotropics, Testosterone, Tropical birds

## Abstract

**Background:**

Birds, across their annual cycle, progress through sequences of life-history stages such as reproduction and molt. The mechanisms that control annual avian itineraries involve endocrine responses triggered by seasonal environmental factors, including changes in resource availability and/or photoperiod. However, at equatorial latitudes birds are exposed to different degrees of seasonality, and the mechanisms underlying phenology of birds near the equator remain less explored. We studied the silver-beaked tanager, an endemic Amazonian songbird, from an equatorial lowland population. Remarkably, in this species, song behavior has been shown to be seasonally aligned to minimal changes in day length near the equator. Here, we aimed to further explore the phenology of silver-beaked tanagers by assessing shifts of food sources utilization as potential ultimate factors. We measured triple isotopic tracers of carbon (δ^13^C), nitrogen (δ^15^N) and sulphur (δ^34^S) in blood and feathers of birds throughout a whole year. In addition, we assessed the degree of seasonality in the molting activity, in relation to circulating levels of corticosterone, as well as to testosterone as a proxy of the reproductive condition of males.

**Results:**

There was important seasonal variation of δ^34^S values in relation to rainfall patterns and changes in estuarine water composition. Despite the seasonal rainfall, we found no substantial variation in the foraging ecology of birds over seasons. This was accompanied by uniform levels of corticosterone throughout the year, probably associated with the absence of drastic seasonal resource shortages. Even so, silver-beaked tanagers showed a marked seasonal molting schedule, which was related to variation in the circulating levels of both corticosterone and testosterone.

**Conclusions:**

These findings suggest that foraging niche is not life history stage-dependent in silver-beaked tanagers, and highlight rainfall as an important environmental cue for bird phenology. Our stable isotope results encourage further studies addressing the influence of estuarine water dynamics on bird timing. In addition, the results suggest a primary role of steroid hormones in regulating seasonal life history stages under the absence of a marked photoperiod. Contrary to what might be expected for a tropical songbird, our physiological data in silver-beaked tanagers do not support reproduction-molt overlapping.

**Electronic supplementary material:**

The online version of this article (10.1186/s12983-018-0284-7) contains supplementary material, which is available to authorized users.

## Background

Animals typically undergo seasonal changes in morphology, physiology and behavior aligned with the cyclical fluctuations of the environment. The seasonal timing of recurring biological processes is known as phenology [1, 2]. Since the seminal studies of Rowan (1925, 1926), it has been well known that changes in annual day length (photoperiod) operate as a primary cue timing avian phenology [3, 4]. In temperate and arctic regions the increasing photoperiod usually predicts the advent of favorable weather and increasing food availability, which are thought to be important ultimate factors for the seasonal timing of energetically demanding life-history stages, such as breeding and molt [5]. In contrast, marked photoperiodic cycles are absent for birds species living near the equator. Field and experimental data suggest nonetheless that equatorial avian species express well defined seasonal itineraries of activity [6, 7], including in Amazonian [8–10], which may rely on a robust endogenous rhythmicity [11–13]. It has been proposed that even minimal photoperiodic changes near the equator can be exploited by birds [14, 15]. Elsewhere it is shown that tropical birds generally encounter relatively lesser seasonality in the environment than temperate species, permitting relatively longer breeding seasons, with more tropical species exhibiting arrhythmic or aseasonal breeding [9, 16]. However, we still have only a vague idea of how equatorial species align their phenology with the local environment.

At a proximate level, the steroid hormones, including testosterone and corticosterone, are important physiological mediators of the avian phenology [17, 18]. Seasonal production of gonadal testosterone in males influences territorial and courtship behaviors, and the activation of different sexual traits [19–22]. The increase of the testis size of males during the reproductive stage is typically accompanied by a seasonal peak of circulating testosterone levels [23], which support physiological and behavioral processes that enhance fecundity of males, including tropical avian species [24, 25]. On the other hand, changes in corticosterone (the main glucocorticoid in birds) in plasma, at baseline levels, are involved in the modulation of the energy balance, and might regulate energy intake, storage and mobilization [26–28]. Glucocorticoids are vital mediators of the physiological and behavioral responses of vertebrates to energetic and environmental demands [29]. For instance, birds respond to food restriction or low quality food with increasing baseline corticosterone levels [30–33], and this rise mobilizes energy to maintain homeostasis [34]. Thereby, corticosterone levels are thought to track the body’s energy requirements, and adjust systemic responses through different life history stages of birds [17]. Yet, research continues to be biased toward temperate zone birds of the northern hemisphere. This bias limits our understanding about the control of phenology at different contexts, and impedes physiological comparative analysis across species with different evolutionary histories, such as those residing in the equatorial regions.

The silver-beaked tanager (*Ramphocelus carbo*) is an endemic songbird of the Amazon biome, whose genus *Ramphocelus* consists entirely of species distributed throughout the tropics [35]. We have shown previously that male silver-beaked tanagers perform a dawn-song behavior seasonally, aligned to minimal increases in day length [15, 36]. In addition, as described for several tropical species, silver-beaked tanagers have an extended reproductive period, evidenced by the long maintenance of their breeding territories and dawn-song behavior [15, 36]. Based on this reproductive seasonality of silver-beaked tanagers in equatorial eastern Amazonia, we aim to further explore the factors governing their phenology by assessing molt timing associated with steroid hormone levels, and the variation in the use of dietary sources over a year.

We studied silver beaked tanagers in an estuarine region of the Amazon near the equator. The inflow of sea and fresh water of estuaries provides large amount of nutrients in the sediment and soil, making estuaries highly productive ecosystems [37]. Silver-beaked tanager are non-migratory, omnivorous birds [8, 10, 38]. Thus, we hypothesize that if birds experience important seasonal shifts in their foraging niche, this shift could function as a potential ultimate factor for the timing of their reproductive and molt phenology. To test this assumption, we measured triple-stable isotopes tracers of carbon (δ^13^C), nitrogen (δ^15^N) and sulphur (δ^34^S) in blood and feathers of males, two tissues that differ in their isotopic turnover, throughout different time points of the year. The δ^15^N ratios of animals estimate changes in trophic levels, since the δ^15^N of a consumer is typically enriched relative to its prey [39]. The δ^13^C ratio is an indicator of diets based on plants with distinct photosynthetic pathways (e.g., C3 vs. C4) [40, 41]. Given the estuarine contexts, we also used δ^34^S signatures as indicator of marine influences on diet, since marine-derived sulfates are generally more enriched in δ^34^S than terrestrial sources [42].

Additionally, we tracked the phenology of molt in males, in terms of seasonality and synchronicity, and assessed its relationship with steroid hormones levels. Tropical avian species, in general, are thought to have the capacity to regularly molt and breed simultaneously [16, 43], however few studies have addressed the environmental and physiological control of molt phenology in equatorial birds. In general, little is known about the physiological basis regulating transitions of life history stages in equatorial species.

Hence, while studying the seasonality of feeding and molting, we assessed the seasonal production of circulating corticosterone and testosterone. Male silver-beaked tanagers express seasonal levels of testosterone in plasma that correlate positively with changes in testis size [36]. Thus, higher levels of testosterone represent a good physiological indicator for gonadal and reproductive activity of male silver-beaked tanagers. On the other hand, as this species inhabits a highly productive environment, we expect that individuals may express low variability in their levels of baseline corticosterone over seasons, as a response to relatively stable conditions that they might experience during the year.

## Methods

### Subject, study site and field procedures

The silver-beaked tanager (*Ramphocelus carbo*) is a non-migratory, sexually dimorphic species [8, 10, 38]. It belong to the passerine family Thraupidae, which comprises 12% of the Neotropical avifauna [44]. Male silver-beaked tanagers typically display seasonal dawn-song that is directly involved in the establishment of breeding territories [36].

The study site was located approximately 60 km to the northeast of the city of Belém in Brazil (1°12′07″S 48°18′07″W, 30 m above sea level) in the Amazon River basin. The annual variation in day length is ca. 9 min (on-line day length calculator; United States Naval Observatory, Astronomical Applications Department). According to the Köppen climate classification [45] the region has an equatorial rainforest climate, which presents annual average temperature of 26 ± 4 C°. Although there is no strictly defined “dry” season because it rains almost every day, there is however a distinct rainy season with very high daily rainfall (between December and May); while the drier season lasts from June until November [46, 47]. The precipitation levels of our study site during the year of sampling were obtained daily from the Instituto Nacional de Meteorologia website (INMET; www.inmet.gov.br).

Individual males were caught using mist nests and playback of conspecific songs. The sampling schedule for steroid hormones and molt registers was divided in five monthly periods: April, July, and November of 2011, January and February of 2012. Within 3 min post capture, a blood sample from the wing vein was obtained in 57 males to determine baseline corticosterone. Then, within 5 min after capture a second blood sample was obtained to determine testosterone levels from 109 males. Blood samples were collected using heparinized capillaries and stored on ice until return to the field station to be centrifuged, then the plasma was separated and frozen (− 40 °C). After the blood sample was taken, birds were weighed and checked to be molting and released. We classified individuals as molting when at least one of the flight feathers (tail and wings) were symmetrically molting.

**Fig. 1:**
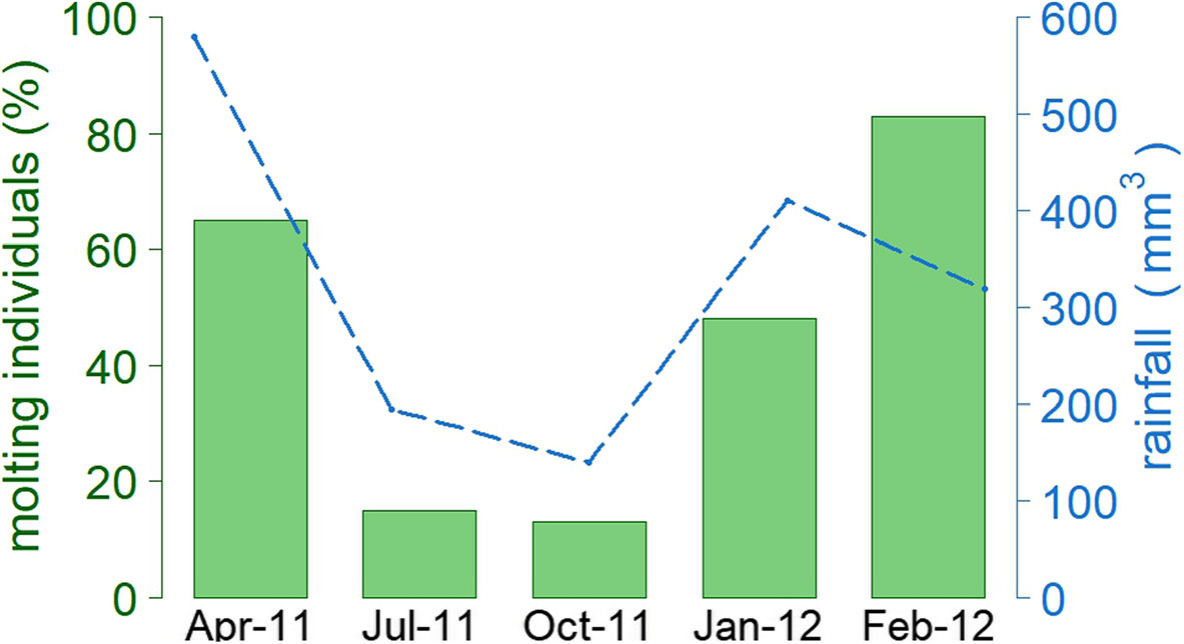
Annual molt activity follows seasonal rainfall patterns. Barplot shows percentage of individuals that were molting within the total of individuals caught during each of sampling periods. The blue line indicates the rainfall in mm during each period

For the stable isotope analyses in blood cells, we collected blood samples of different males during four monthly periods: April, July, and October of 2011 and January 2012. Seven blood samples were obtained during each period (28 in total). Once collected, blood samples were kept frozen (− 40 °C) until analysis. In addition, for the stable isotope analyses of feathers, we collected one of the central tail feathers, or rectrices. We obtained 15 feathers from selected non-molting adult males on July 2011, with rectrices that seemed recently molted, clearly distinguished by their smooth edges and good shape; and therefore presumably grown late in the molting season, around April–May, during the rainy season (see Fig. 1). On the other hand, in February 2012 during the molt season, we collected 14 feathers from selected molting males, but with already newly grown central rectrices. Therefore these feathers were presumably grown at the beginning of the molting season, around December–January, at the end of the dry season (see Fig. 1). Stable isotope signatures obtained from blood cells provides short to medium term information (about 2–3 weeks before sampling time), while feathers reflect the diet at the time when they have grown [48–50].

### Hormone analysis

Testosterone and corticosterone concentrations were determined by radioimmunoassay following the procedures described in [51]. Samples were assayed in duplicate and distributed randomly between two assays. The extraction recovery for testosterone was 88.0% ± 2.8% (mean ± sd). Hormone concentrations were calculated with Immunofit 3.0 (Beckmann Inc., Fullerton, CA, USA). The lower detection limits of assays were 0.37 pg/ml and 0.40 pg/ml tube, respectively, and all samples were above the detection limit. The intra-assay coefficients of variation were 9.1% and 4.2%, respectively; the intra-extraction coefficients of variation of a chicken plasma pool were 3.3% and 6.0%, respectively. The inter-assay coefficient of variation between the two assays was 9% and the inter-extraction coefficient of variation between the two assays was 15.9%. For corticosterone, samples were assayed in duplicate in one assay. The extraction recovery was 79% ± 3.9% (mean ± sd), the lower detection limit of the assay was 4.14 pg/ml and all samples were above the detection limit. The intra-assay coefficient of variation was 4.8%, and the intra-extraction coefficient of variation of a chicken plasma pool was 9.0%.

### Stable isotopes analysis

We measured stable isotope ratios for carbon (δ^13^C), nitrogen (δ^15^N), and sulfur (δ^34^S) in blood cells and feathers. Feather samples were rinsed with methanol and air-dried in a fume hood. The stable isotope analysis were conducted on sub-samples of approximately 0.7 mg cellular blood (plasma free), and 0.5 mg feather were weighed into small tin cups to the nearest 0.001 mg, using a micro-analytical balance. Dried, powdered samples were loaded into tin capsules and combusted in a vario Micro cube elemental analyzer (Elementar, Analysen systeme, Germany). The resulting gases were fed via gas chromatography into the inlet of a Micromass (Manchester, UK) Isoprime Isotope Ratio Mass Spectrometer (IRMS). Measurements are reported in δ-notation in parts per thousand deviations (‰) relative to international standards for carbon (Pee Dee Belemnite, PDB) and nitrogen (atmospheric N_2_, AIR, S0_2_), according to the equation δ (‰) = 1000 x (Rsample/Rstandard-1).

Two sulfanilamide (Iso-prime internal standards), and two Casein were used as a laboratory standard for every 10 unknowns in sequence. The reference material used for sulphur isotope analysis was sulfanilamide calibrated and traceable to NBS-127 (barium sulphate, δ^34^S = + 20.3 ‰). Replicate assays of internal laboratory standards indicate measurement errors (SD) of ±0.05‰, 0.15‰ and 0.05‰ for δ^13^C, δ^15^N and δ^34^S, respectively.

### Statistical analysis

R version 3.2.0 (R Development Core Team 2015) was used for all statistical analysis. Stable isotope data were analyzed using general linear models. We checked whether stable isotope (δ^15^N, δ^13^C, δ^34^S) values in blood changed over time with months of the year as explanatory variable (April, July, November of 2011 and January of 2012). Changes in the isotopic values of feathers were analyzed over two sampling periods (July 2011 and February 2012).

Hormone levels in plasma were analyzed using general linear models. Changes in circulating levels of testosterone over five time points (April, July, November of 2011, and January and February of 2012) were tested with body mass, molting condition, sampling hour, and corticosterone levels as covariates; testosterone data were log transformed to meet normality assumptions. Additionally, changes in baseline levels of corticosterone in plasma over seasons (April, July, November of 2011, and January and February of 2012) were tested with body mass, molting condition, sampling hour, and testosterone levels as covariates; corticosterone data were log transformed.

Besides, in order to visualize the relationship between corticosterone levels and daytime of sampling, a correlation test was performed using Pearson analysis (Additional file 1).

## Results

### Stable isotopes profiles

**Fig. 2:**
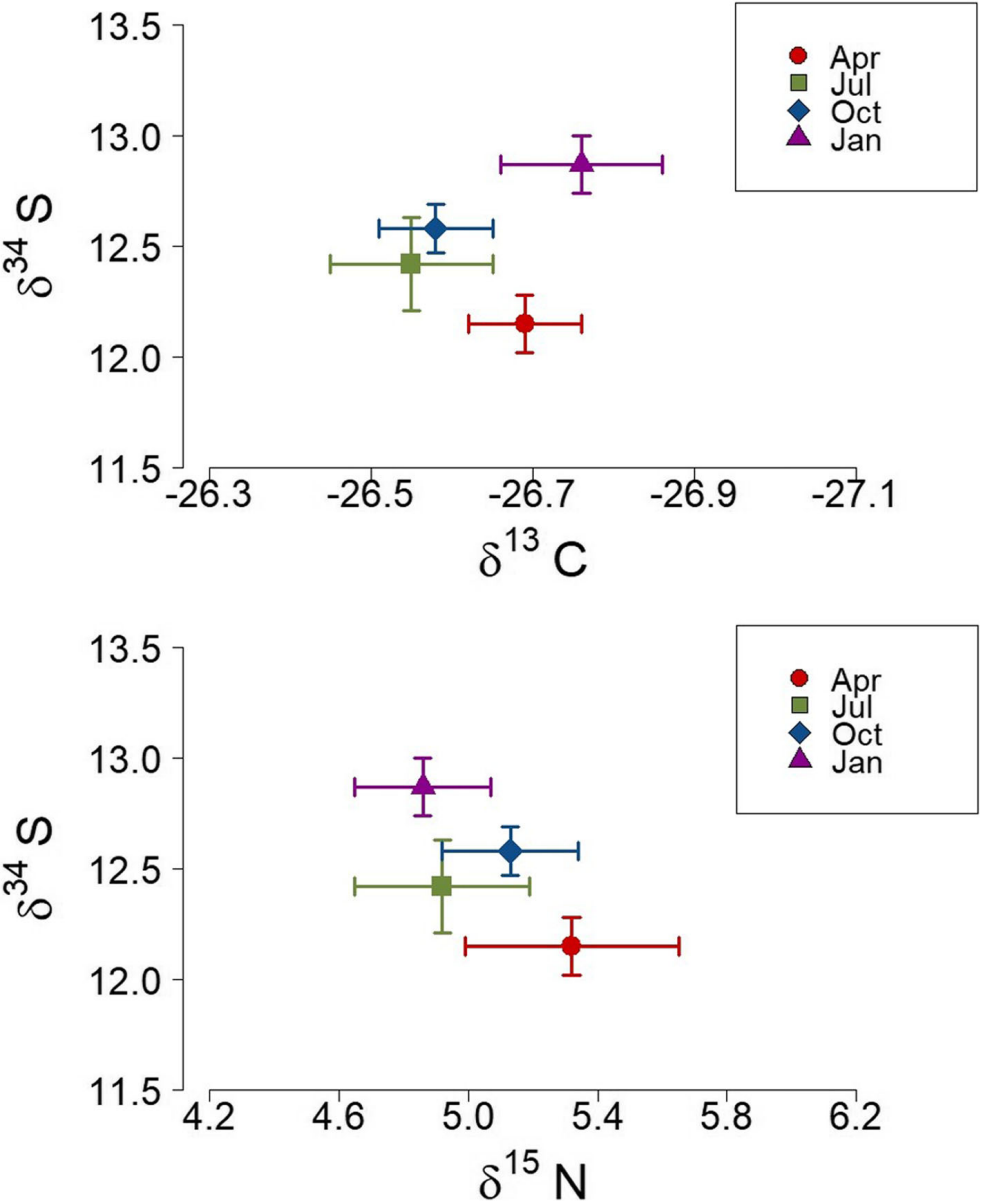
Bi-plot of stable carbon, nitrogen and sulfur isotope values for blood cells indicates no major changes in the foraging ecology over the year, but seasonal variations in the sulfur signatures. The symbols represent the mean values (+/− SE) over four sampling periods (April, July, October 2011 and January 2012)

**Fig. 3:**
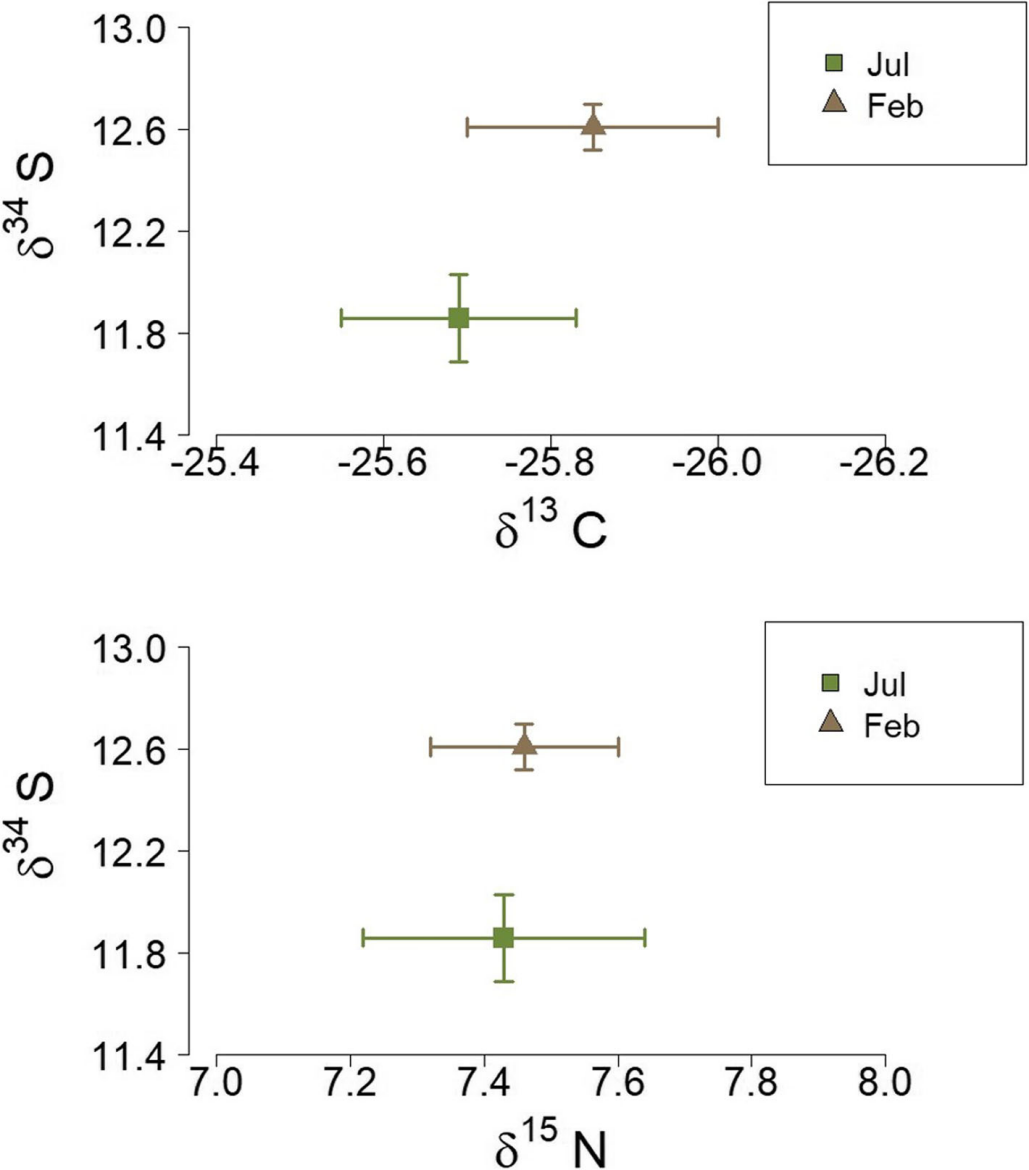
Bi-plot of stable carbon, nitrogen and sulfur isotope values for feathers indicates no major changes in the foraging ecology over the year, but seasonal variations in the sulfur signatures. The symbols represent the mean values (+/− SE) obtained over two sampling periods (July 2011 and February 2012)

We found no seasonal changes of δ^15^N in blood cells (*F*_3,23_ = 0.6, *p* = 0.6) and feather (*F*_1,27_ = 0.009, *p* = 0.9). Also, no seasonality of *δ*^13^C was recorded in blood cell (*F*_3,23_ = 1.24, *p* = 0.32) and feather (*F*_1,27_ = 0.55, *p* = 0.46), (Figs. 2 and 3). However, δ^34^S signatures in blood cells were statistically higher in January 2012 relative to April 2011 (Fig. 2) (*F*_3,23_ = 3.8, *p* = 0.02). Similarly, feather *δ*^34^S values changed significantly throughout the seasons, with values significantly higher in February 2012 relative to July 2011 (*F*_1,27_ = 14.8, *p* = 0.0006), (Fig. 3).

### Hormonal profiles

**Fig. 4:**
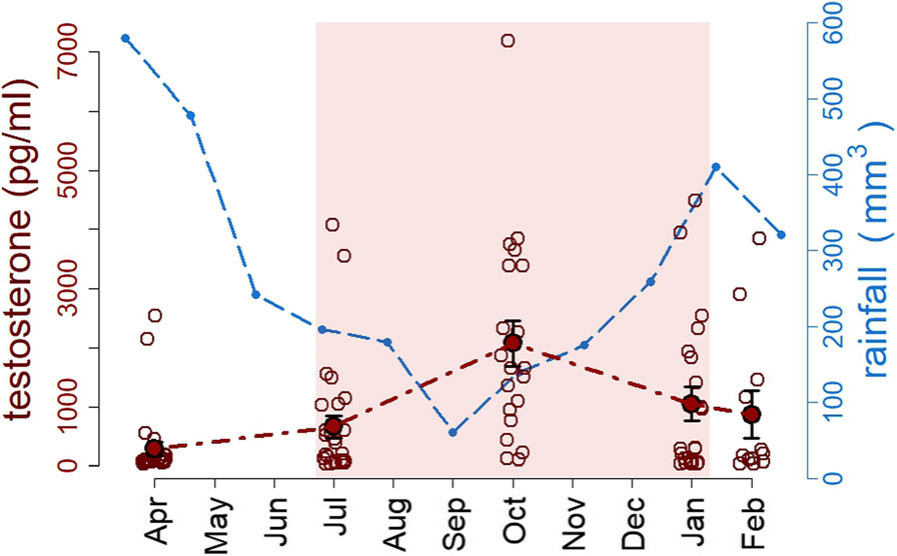
Seasonal changes of testosterone levels in males. The seasonal peak of testosterone occurs during the dry season. The red line and filled circles shows means (+/− SE) of testosterone in males over the five sampling periods (April, July, October 2011, and January, February 2012). Red open circles indicate male individual testosterone levels. The blue line indicates the rainfall in mm represented as the monthly values from April 2011 until February 2012. The red shading indicates the period during which seasonal dawn-song occurs [36]

**Fig. 5:**
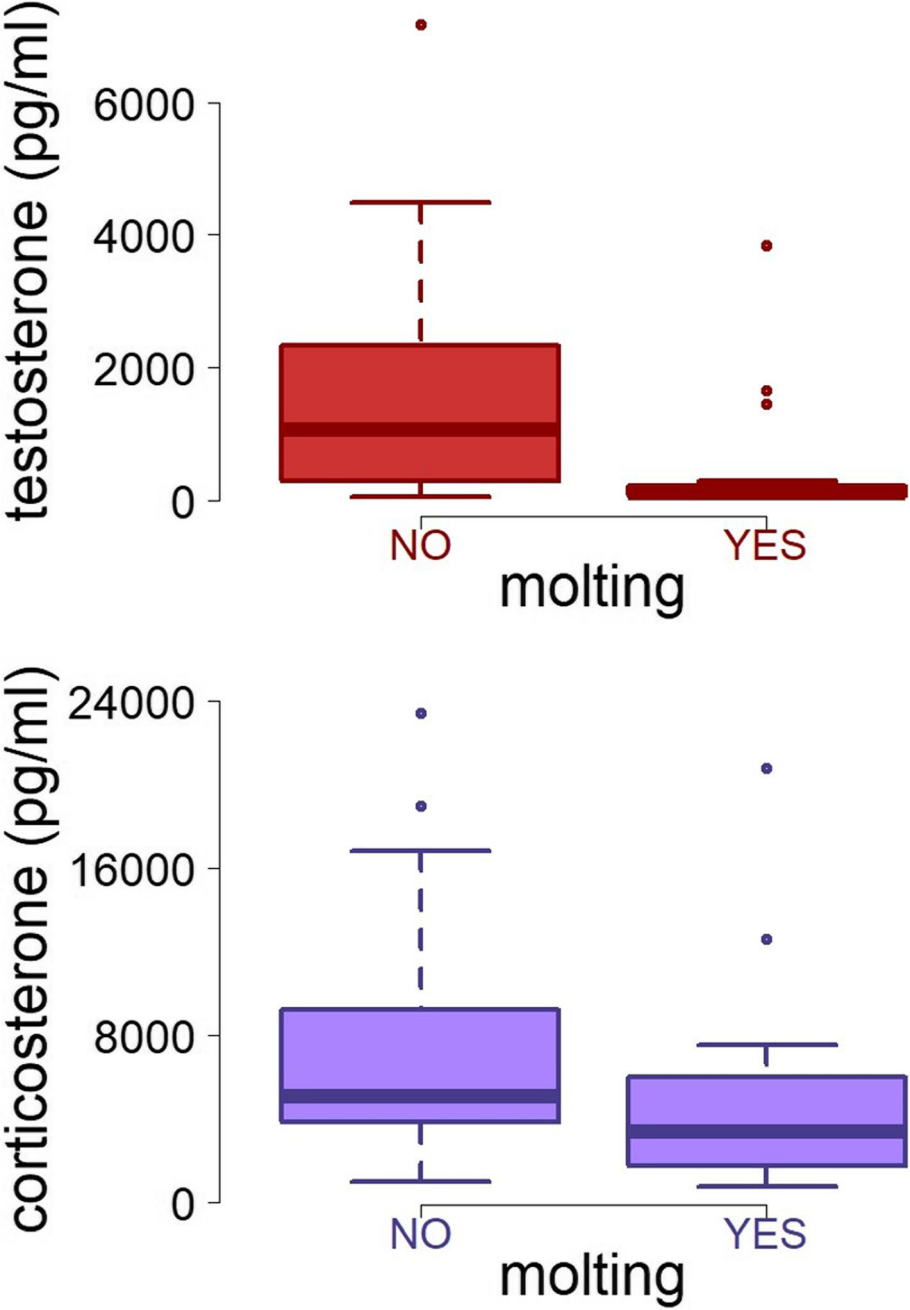
Individuals present low levels of testosterone and corticosterone during molt. The upper and lower panels show circulating levels of testosterone and baseline levels of corticosterone respectively, in relation to individual molt conditions of males. Box plots indicate medians, 10th, 25th, 75th, and 90th percentiles, dots indicate individual outlier values. Hormonal values were obtained over five different time points throughout the year

There was a significant effect of season on plasma testosterone. Testosterone levels were significantly higher in November, January 2011 and February 2012 compared to April 2011 (*F*_4,97_ = 6.3, *p* = 0.003) (Fig. 4). Further, molt and testosterone were significantly related (*F*_1,97_ = 23.7, *p* < 0.0001): males with higher concentrations of circulating testosterone were not undergoing molt (Fig. 5). In addition, the model indicated a marginally significant negative influence of sampling hour (hour of the day) on testosterone levels (*F*_1,97_ = 6.0, *p* = 0.044). Body mass and testosterone were not related (*F*_1,97_ = 0.2, *p* = 0.66). There was no effect of baseline corticosterone on the testosterone levels (*F*_1,97_ = 2.4, *p* = 0.12).

**Fig. 6:**
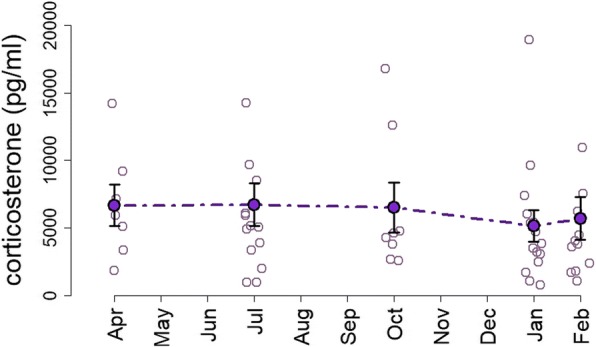
Uniform baseline levels of corticosterone over the year. The purple line and filled circles shows means (+/− SE) of baseline levels in males obtained over the five sampling periods (April, July, October 2011, and January and February 2012)

Molt was significantly related to baseline corticosterone (*F*_1,49_ = 9.8, *p* = 0.003), with molting males expressing lower levels of corticosterone than non-molting males (Fig. 5). However, we found no effect of the month of sampling (season) on baseline corticosterone concentrations (Fig. 6) (*F*_4,49_ = 1.0, *p* = 0.41). Also, no significant effects were observed for body mass (*F*_1,49_ = 0.3, *p* = 0.55) or for testosterone levels on baseline corticosterone (*F*_1,49_ = 1.5, *p* = 0.216). Furthermore, the model showed a significant effect of the sampling hour on circulating corticosterone levels (*F*_1,49_ = 8.0, *p* = 0.006). Baseline corticosterone significantly decreased from morning to evening hours (Additional file 1).

## Discussion

### Isotopic values and seasonal estuary dynamic

δ^13^C and δ^15^N values were used as proxies of the birds’ foraging habitat and diet, respectively, and were measured in two tissues (blood cells and feathers) that record trophic information at different time scales [49, 52]. The results suggest that foraging niches might not be life-history stage-dependent, with males depicting no pronounced temporal shift in their feeding sources (δ^13^C and δ^15^N) (Figs 2, 3). The study provides further evidence that tropical birds are not exposed to extreme changes in food accessibility, and they would not have need of drastic shifts in trophic levels [39–41]. Minor fluctuations in temperature, daily occurrence of rain (even during the dryer season), and high nutrient deposition of the Amazon estuary may permit a constant availability of fruits as well as insects for birds over the year. Although we are not able to directly infer seasonal changes in food abundance from the isotopic analysis, the invariant levels of baseline corticosterone showed by birds suggest they are not exposed to periods of drastic food shortages or restricted access to food resources. Overall, these results indicate that seasonal shifts in the foraging niche do not represent a significant ultimate factor for the timing of life history stages in silver-beaked tanagers.

Interestingly, however, there was strong seasonality in δ^34^S values in both tissues (Figs. 2, 3). The δ^34^S signatures are used as indicator of marine influences on feed sources, since oceanic-derived sulfates are generally more enriched in δ^34^S than terrestrial sources [42]. However, δ^34^S values do not necessarily reflect direct consumption of marine sources, but can reflect the proximity of soil and food sources to the sea [53]. The Amazon estuary is a wide transition zone, subject to marine and fluvial influences, in which silver-beaked tanagers have permanent access to extensive and winding riparian areas to search for food. Therefore, δ^34^S signatures of birds might indicate seasonal changes in the composition of mixed water. In fact, the highest δ^34^S values obtained in blood cells (January) correlate with a period of lower freshwater discharge, whereas the lowest δ^34^S values (April) coincide with the period of greater fluvial discharge in the region of the study [54, 55] (Fig. 1). Similar results were obtained with feathers, where the highest δ^34^S values were obtained in feathers presumably grown in December – January, whereas the lowest values were obtained from feathers presumably grown in April–May (Fig. 3). Alternatively, δ34S ratios could also depict changes in the quality of the dietary protein, in relation to the intake of food items rich in sulfur amino acids, such as methionine, cystine, cysteine and taurine [42, 56]. Further studies that precisely examine foodweb values, and the dietary items ingested by birds throughout the year are required to prove this possibility.

These results provide insight into the potential significance of seasonal dynamics of estuaries as an environmental factor. It could be that changes in the salinity, and mixed water composition of the Amazon estuary, associated with the seasonal rainfall regimes, function as a relevant supplementary cue to fine-tune phenology of silver-beaked tanagers. We hope this work encourages future research efforts to assess how seasonal estuarine dynamics interact with the life history of birds; and how birds pick signals to respond to the changing environment in equatorial Amazonia.

### Seasonal and diurnal variations in steroid hormones

The gonadal testosterone production of males were increased during the dry season, which supports the assumption that male silver-beaked tanagers might undergo seasonal reproductive cycles (Fig. 4). In addition, the results suggest that testosterone levels tend to have a diel rhythm of secretion, as has been proposed for other avian species from higher latitudes [57–59]. In tropical stonechats the seasonal peak of testosterone levels occurs during the nest building, which correspond to the period of higher territoriality and aggressive responses of males to conspecifics [60]. Accordingly, male silver-beaked tanagers display a territorial dawn-song behavior in a seasonal manner, with an extended reproductive period that coincides with the dry season [15]. Moreover, at our equatorial study site birds experience a photoperiodic variation of approximately 9 min throughout the whole year. The levels of circulating testosterone of males peaked through October and November (Fig. 3), about three months following winter solstice [36]. Therefore, some tropical birds seem to use minimal changes in photoperiod to time seasonal reproductive activities [14, 15].

No seasonality was found in plasma levels of baseline corticosterone (Fig. 6), although there was an influence of day time on the baseline corticosterone level. Natural production of glucocorticoids play an important role in the energetic metabolism of birds. Thus, circulating levels of glucocorticoids are adjusted on both a seasonal and a daily basis according to predictable fluctuations in the balance between the energetic demands and the energy available in the environment [26, 61–64]. Here, the results suggest that males present seasonally uniform levels of corticosterone, which might be associated with the stable availability of dietary resources, and the absence of drastic energetic restrictions over seasons [65]. Accordingly, our isotope analysis suggest no pronounced shift in the use of trophic resources throughout a year. On the other hand, the results indicated an important effect of day time on the baseline levels of corticosterone in males. Individuals sampled early in the morning had a significant tendency to express higher corticosterone levels, and the levels tend to decline toward the later hours of the day (Additional file 1). Diel rhythm of corticosterone mediate the initiation of daily activity and foraging onset in birds [66]. Thus, these results suggest that corticosterone plays a primary role at a diurnally scale, in mediating physiological and behavioral daily processes of birds.

### Seasonal variation in molt activity

The silver-beaked tanager exhibited a marked periodicity in feather replacement, whereby most male birds predominantly molt during the rainy season and displayed a marked synchronicity in their molting timing (Fig. 1). At the individual level, molting males presented significantly lower concentrations of circulating testosterone than non-molting males (Fig. 5). These results suggest that similar to temperate zone species, silver-beaked tanagers avoided the overlap of breeding and molt [67], since high testosterone levels are incompatible with the molting activity of males. This outcome suggests the existence of a physiological trade-off between the regulation of breeding and molt, in which a high production of gonadal testosterone is directed to facilitate reproductive functions, but is unfavorable for the requirements of the molt stage. It is likely that the occurrence of molt in male silver-beaked tanagers was activated by the termination of the reproduction and the decreasing levels of gonadal testosterone. On the other hand, individuals that were molting exhibited comparatively lower levels of baseline corticosterone (Fig. 5). This seems to be related to a proteolytic effects of glucocorticoids, which limit the availability of critical amino acids required for feather growth [67, 68]. These results support the idea of steroid hormones as important mediators of the reproduction-molting cycle of silver-beaked tanagers tanagers, which seems to be controlled by endocrine regulatory tradeoff, given that both stages are energetically demanding.

## Conclusions

Few studies have addressed the regulation of life history stages in the annual cycle of equatorial birds (Apfelbeck et al., 2017, [60]; Goymann et al., 2012, [12] , Goymann et al. 2006, [51] Gwinner and Scheuerlein, 1998 [69]; Moore et al., 2005 [70]), and even fewer in species of the Amazon rainforest. Our results suggest that silver-beaked tanagers do not undergo major shifts in their foraging ecology throughout the year. This conclusion was supported by the uniform levels of baseline corticosterone exhibited by silver-beaked tanagers throughout seasons, which might indicate unconstrained conditions in terms of energetic requirements. The peak in the molting activity of males occurred along with the seasonal decrease of rainfall (Fig. 1), in which both testosterone and corticosterone were lower in molting individuals (Fig. 5). Contrary to what is traditionally thought for tropical birds, our physiological data do not support a reproduction-molt overlap in equatorial silver-beaked tanagers. Overall, the phenology of birds at this equatorial Amazonian region seems to follow the annual photoperiod, despite the low amplitude of the photoperiodic cycle [15, 36]. Further, the end of the rainy season and the change in the water composition in the estuary may serve as supplementary environmental signals for silver-beaked tanagers. Supplementary cues are regularly used by other birds from tropical environments to fine-tune phenology [3, 11, 71–73]. Our results underline the relevance of studying hormonal proximate factors in combination with environmental ultimate causes for a better compression of life history stage transitions in equatorial species.

## Additional file

Additional file 1: Diel levels of corticosterone are high during the morning and tend to decrease towards the later hours. The scatter plot shows a negative correlation between log-transformed levels of baseline corticosterone and the hour of the day when the sample was taken (*r* = − 0.35, *p* = 0.009). The samples were collected over the course of five sampling periods throughout the year. (JPG 57 kb)
