# Supplementary figures and images for: Seasonality at the equator: isotope signatures and hormonal correlates of molt phenology in a non-migratory Amazonian songbird
Source: Front Zool. 2018 Oct 29;15:39. doi: 10.1186/s12983-018-0284-7 (PMC6205779; doi:10.1186/s12983-018-0284-7)

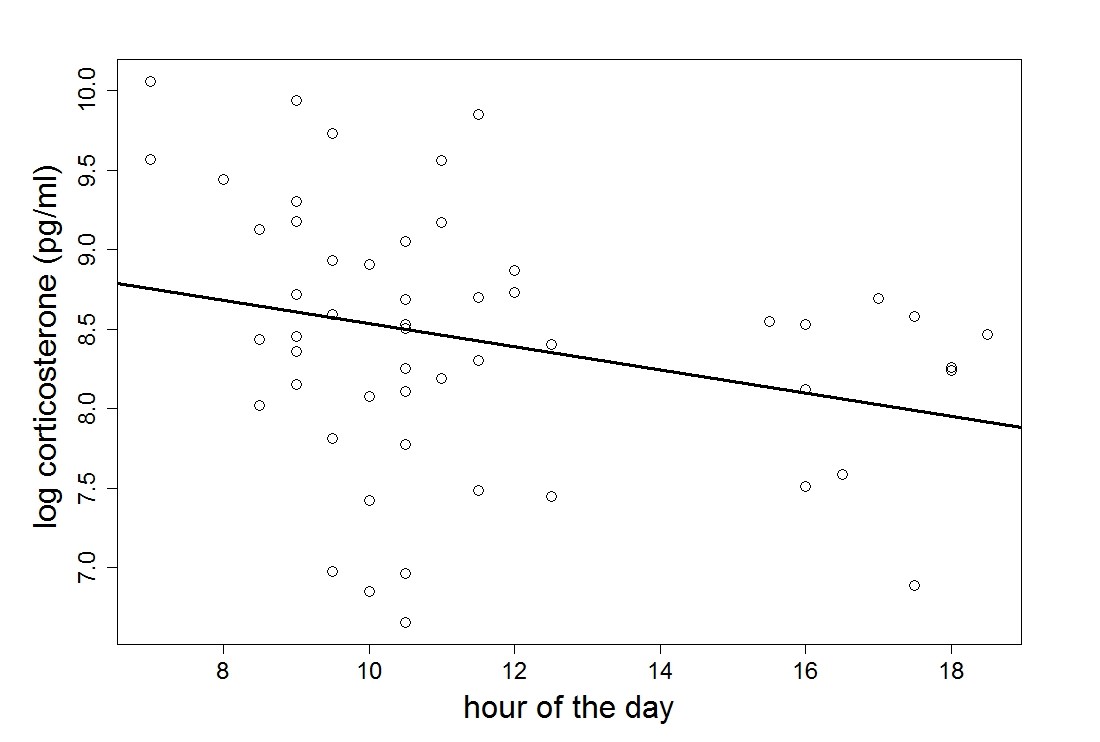

Supplement: Supplementary file 1 — Diel levels of corticosterone are high during the morning and tend to decrease towards the later hours. The scatter plot shows a negative correlation between log-transformed levels of baseline corticosterone and the hour of the day when the sample was taken (r = − 0.35, p = 0.009). The samples were collected over the course of five sampling periods throughout the year. (JPG 57 kb) [file 12983_2018_284_MOESM1_ESM.jpg]
